# Photosynthetic adaptation strategies in peppers under continuous lighting: insights into photosystem protection

**DOI:** 10.3389/fpls.2024.1372886

**Published:** 2024-05-31

**Authors:** Jason Lanoue, Sarah St. Louis, Celeste Little, Xiuming Hao

**Affiliations:** Harrow Research and Development Centre, Agriculture & Agri-Food Canada, Harrow, ON, Canada

**Keywords:** continuous lighting, chlorophyll fluorescence, carbohydrates, reactive oxygen species, circadian rhythm, dynamic 24h lighting

## Abstract

Energy efficient lighting strategies have received increased interest from controlled environment producers. Long photoperiods (up to 24 h - continuous lighting (CL)) of lower light intensities could be used to achieve the desired daily light integral (DLI) with lower installed light capacity/capital costs and low electricity costs in regions with low night electricity prices. However, plants grown under CL tend to have higher carbohydrate and reactive oxygen species (ROS) levels which may lead to leaf chlorosis and down-regulation of photosynthesis. We hypothesize that the use of dynamic CL using a spectral change and/or light intensity change between day and night can negate CL-injury. In this experiment we set out to assess the impact of CL on pepper plants by subjecting them to white light during the day and up to 150 µmol m^-2^ s^-1^ of monochromatic blue light at night while controlling the DLI at the same level. Plants grown under all CL treatments had similar cumulative fruit number and weight compared to the 16h control indicating no reduction in production. Plants grown under CL had higher carbohydrate levels and ROS-scavenging capacity than plants grown under the 16h control. Conversely, the amount of photosynthetic pigment decreased with increasing nighttime blue light intensity. The maximum quantum yield of photosystem II (F_v_/F_m_), a metric often used to measure stress, was unaffected by light treatments. However, when light-adapted, the operating efficiency of photosystem II (ΦPSII) decreased and non-photochemical quenching (NPQ) increased with increasing nighttime blue light intensity. This suggests that both acclimated and instantaneous photochemistry during CL can be altered and is dependent on the nighttime light intensity. Furthermore, light-adapted chlorophyll fluorescence measurements may be more adept at detecting altered photochemical states than the conventional stress metric using dark-adapted measurements.

## 1 Introduction

Light is the driving force for carbon assimilation in plants, however there is a species-specific desired/optimum daily light integral (DLI – photoperiod x light intensity) – an excessive or deficient amount can impact plants negatively. Too much light can be harmful to plants as it significantly reduces the efficiency of photosynthesis which can lead to photoinhibition causing damage to photosystem II (PSII) (Barber and Andersson, 1992). Long photoperiods can also be harmful to plants. While the photoperiodic threshold is different for each plant species, generally, photoperiods longer than 17h can cause leaf damage observed as interveinal chlorosis and decreased maximum quantum efficiency of PSII (F_v_/F_m_); an indicator of stress (Baker, 2008; Sysoeva et al., 2010; Velez-Ramirez et al., 2011).

Theoretically, the implementation of CL strategies can increase yield if photoperiod-related injury is averted (Velez-Ramirez et al., 2012). While some crops are tolerant to CL (Ohtake et al., 2018; Lanoue et al., 2021b), others, such as pepper, are observed to have altered leaf shape, chlorosis, and reductions in yield when compared to peppers grown under shorter photoperiods (Demers et al., 1998b; Demers and Gosselin, 1999). Lengthening the photoperiod can also lead to reduced stem elongation in peppers (Demers et al., 1998a) resulting in fruit being too close together and misshapen which negatively impacts fruit quality (Lanoue et al., 2022b). It is therefore important to identify long photoperiod (including CL) strategies which can overcome reductions in stem elongation and maintain adequate fruit quality.

The underlying mechanism of CL-injury is unknown. Current hypotheses include a mismatch between the endogenous circadian rhythm and exogenous environmental cues (Velez-Ramirez et al., 2017b; Marie et al., 2022), improper gene expression (Velez-Ramirez et al., 2014; Inoue et al., 2018), and over-accumulation of photosynthetic products leading to feedback inhibition (Velez-Ramirez et al., 2017a; Pham et al., 2019). Exposure to CL means plants are under constant photon pressure which will continuously drive photosynthesis if the light level is above the light compensation point. With constant photosynthesis comes continuous production of photosynthetic products such as soluble sugars and starch (Globig et al., 1997; Matsuda et al., 2014; Pham et al., 2019). This accumulation of carbohydrates is linked to chloroplast membrane damage, inevitably causing a downregulation of photosynthesis via feedback loops caused by over-reduction of the electron transport chain components (Foyer et al., 2012; Zhu et al., 2020). Consequently, many believe that the buildup of photosynthetic products during CL will impact gene expression and ultimately reduce photosynthesis leading to a reduction in light-use-efficiency (Pammenter et al., 1993; Van Gestel et al., 2005; Smith and Stitt, 2007; Stitt et al., 2010). Our recent research has shown that tomatoes grown under CL with 50 µmol m^-2^ s^-1^ of blue light during the night had similar carbohydrate patterns and levels as those grown under a 16 h control with 8 h darkness (Lanoue et al., 2019). However this light intensity was around the light compensation point and did not drive high rates of photosynthesis which could cause feedback inhibition. Conversely, tomatoes grown under CL with a constant 147 µmol m^-2^ s^-1^ of white light for 24 h showed elevated fructose, sucrose, and starch levels at the end of the subjective night compared to a 16 h control treatment (Haque et al., 2015). The elevated carbohydrate status corresponded with a reduction in F_v_/F_m_ values indicating CL-injury. This suggests that a higher nighttime light intensity without a change in spectrum can raise the carbohydrate levels in plants that are associated with CL-injury.

In addition, the use of CL can significantly increase the amount of photo-oxidative stress a plant is subjected to. Reactive oxygen species (ROS) are a normal by-product of photosynthesis, but when produced in higher quantities during periods of high or prolonged light (such as CL) they can become harmful to the plant. An excess accumulation of ROS can cause severe and irreversible DNA damage resulting in cell death (Huang et al., 2019). In mutated *Arabidopsis* which had reduced antioxidant content (2-Cys peroxiredoxin), plants showed decreased photosynthetic rates during CL compared to wild-type plants (Pulido et al., 2010). Coincidentally, mutated plants also had higher levels of carbonyl groups and hydrogen peroxide in the leaves indicating that a reduction in antioxidant capacity increased ROS concentrations and led to diminished photosynthetic rates (Pulido et al., 2010). ROS can also be used as a signaling molecule to alert the plant to stressful conditions such as high or prolonged light. In this way, a healthy balance between ROS production and scavenging can maintain homeostasis since ROS accumulation can initiate gene expression of detoxifying enzymes (Huang et al., 2019). It has been shown that plants with naturally higher levels of antioxidants and ROS-detoxifying enzymes have less injury when exposed to prolonged photoperiods, even CL (Murage and Masuda, 1997). It is therefore speculated that the ability to scavenge ROS may also play a role in averting CL-injury based on their role in photo-oxidative stress (Kim et al., 2008).

In this study, we set out to identify the impact of different nighttime blue light intensities during CL on the morphology, physiology, and yield of pepper plants. Specifically, we wanted to identify how plant performance (i.e., photosynthesis and chlorophyll fluorescence parameters) would adapt under higher (up to 150 µmol m^-2^ s^-1^) nighttime blue light intensities. Blue light was chosen due to its ability to cause stem elongation when provided as a monochromatic light source (Hernández and Kubota, 2016; Lanoue et al., 2019; Kong and Zheng, 2020). We also chose to measure the carbohydrate metabolism and oxidative stress levels in leaves, since literature suggesting that both carbohydrate accumulation and ROS scavenging ability can play a role in CL-injury. It is hypothesized that underlying biochemicals processes may play an important role in mitigating CL-injury in peppers when exposed to dynamic 24h lighting. Additionally, the traditional stress metric, F_v_/F_m_, may not be the most appropriate measurement to determine plant health/stress or ability to utilize incoming radiation.

## 2 Materials and methods

### 2.1 Plant material and experimental design

Five-week-old pepper (*Capsicum annuum*) cv. ‘Gina’ transplants were planted onto rockwool slabs in a 200m^2^ glass greenhouse at the Harrow Research and Development Centre (Agriculture and Agri-Food Canada, Harrow, Ontario, Canada; 42.03°N, 82.9°W) on September 15^th^, 2021. Plants were trained in a high wire “V” system with 2 stems from each plant at a plant density of 6.0 stems m^-2^. The plants were drip-irrigated as needed using a complete nutrient solution with an electrical conductivity of 2.8 dS m^-1^ and a pH of 5.9. The greenhouse was enriched to 800 µmol mol^-1^ of CO_2_ during both day and night when the greenhouse was not vented. Heating temperature during the day was 21°C with a venting temperature of 25°C. Day humidification set point was 75% with a dehumidification set point of 85%. Nighttime heating temperature was 18°C and venting was 22°C. Night humidification set point was 70% with a dehumidification set point of 85%.

The pepper plants were grown on 6 raised gutters/rows. The rows of plants were separated using light abatement curtains (Obscura 9950 FR W, Ludvig Svensson, Kinna Sweden) which allowed for moisture, air, and heat exchange through the fabric but blocked light transmission. The width of each row is 1.5m. The light abatement curtains were closed during cloudy days and during the night to prevent light treatment contamination. On sunny days, the light abatement curtains were opened to prevent shading of the high intensity solar radiation. Rows on the perimeter served as guard rows throughout the experiment and were not subjected to any lighting treatment. The 4 middle rows were used for lighting treatments. The rows ran in a north-south orientation so that each row can receive same amount of sunlight. Each row was divided into 2 independent experimental plots/units. The length of each plot was 2m (or 2.2m including canopy extension). There were 10 plants or 20 stems per plot. Only the middle 8 plants/16 stems were used for data collection. One plant in each of the 2 ends of the plot was used as guard plant. The 2 stems of each plant was trained into a “V” system, one to the west side and the other to the east side, so that the plants in each plot received sunlight from both west and east side. There was a 1.82m gap between the 2 plots in the same row and light reflection boards were applied to the light fixtures in both ends of each plot to prevent any light contaminations between the 2 plots in each row. The application of 4 lighting treatments to the 4 south plots (first replication/block) and the 4 north plots (second replication/block) was randomized. The lighting treatments in the 2 plots within the same row was different. Therefore, the greenhouse experiment was a randomized complete block design with 2 replications.

**Figure 1:**
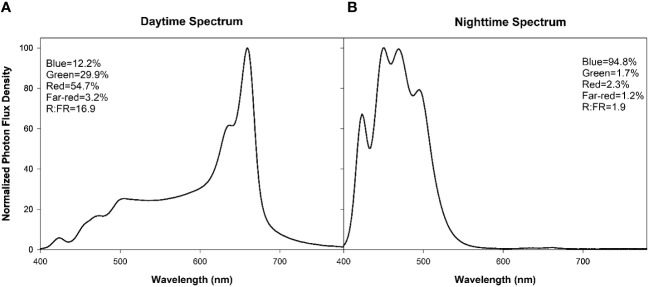
Normalized photon flux density (PFD) of daytime (6:00–20:00, **(A)**) and nighttime (20:00–6:00, **(B)**) light treatments as determined with a Li-180 (Li-COR Biosciences, Lincoln, NE, USA) spectroradiometer during the night at the head of the plant. For each spectrum, the percentages of PFD of blue (400–499nm), green (500–599nm), red (600–699nm), far-red (700–780nm), and the red:far-red (R:FR) are included in the Figure.

The 4 supplemental overhead lighting treatments (0B, 50B, 100B, and 150B, **Table 1**) began on November 3^rd^, 2021. Daytime supplemental lighting was provided by 6 Sollum SF04 multi-channel LED lighting fixtures (Sollum Technologies Inc. Montréal, Québec, Canada) in each plot. Nighttime supplemental lighting was provided by the 6 SF04 smart LEDs or SF04 smart LEDS and RAY66 blue LEDs (for the 150B treatment) from Fluence (Fluence Bioengineering, Austin, Texas, USA) depending on the blue light intensity requirements. Spectral composition readings were taken at the apex of the plant using a Li-COR Li-180 (Li-COR Biosciences, Lincoln, NE, USA) spectroradiometer (**Figure 1**). The daytime white light treatment was applied from 6:00–22:00 (**Figure 1A**) while nighttime blue light treatments, if applicable, were applied from 22:00–6:00 (**Figure 1B**). The light in each treatment was measured at four locations within each plot with a one meter quantum light sensor (Li-COR 191R; Li-COR Biosciences, Lincoln, Nebraska, USA) just above the apex of the plant (**Table 1**). Lights were adjusted as needed to maintain the target light intensity at the apex of the plant throughout the experiment. The total supplemental daily light integral (DLI) was kept similar among all treatments (11.6 ± 0.06 mol m^-2^ d^-1^). All light measurements were performed at night to avoid any contamination from daytime solar radiation. Lights remained on regardless of the natural solar radiation levels to ensure the same total DLIs (sunlight + supplemental light) for all lighting treatments.

**Table 1:** Daytime and nighttime light intensities as measured at four locations within each plot with a one meter quantum light sensor (Li-COR 191R; Li-COR Biosciences, Lincoln, NE, USA) just above the apex of the plant.

Treatment	Daytime (6:00–20:00)Light Intensity (µmol m^-2^ s^-1^)	Nighttime (22:00–6:00) Blue Light Intensity (µmol m^-2^ s^-1^)
0B	200 ± 2	0
50B	175 ± 3	50 ± 1
100B	150 ± 2	100 ± 1
150B	125 ± 2	150 ± 3

Treatment 0B indicates that no light was utilized during the night and that plants in all light treatments were exposed white light spectrum (**Figure 1A**) for 16h from 6:00–22:00. Values represent the average +/- the standard error of light measurements.

### 2.2 Morphological measurements

On January 24^th^, 2022, morphological measurements were performed on eight plants from each treatment. The internode length was determined by measuring the distance between the top of the plant and the tenth node. This distance was then divided by ten to get the average internode length. Leaf length and width of the 5^th^ leaf were measured using a ruler. Stem diameter at the 5^th^ node was measured using a digital caliper. The specific leaf weight (SLW) of the 5^th^ leaf was determined by removing it from the plant, weighing it, and then measuring its leaf area (Li-COR 3100, Li-COR Biosciences Inc. Lincoln, NE, USA). The leaf was then dried in an oven at 70°C. Once dry, the leaf was reweighed. The dry weight was divided by the leaf area to obtain the SLW. Dry matter content was calculated by dividing the dry weight by the fresh weight then multiplying by 100.

### 2.3 Leaf gas exchange

On January 25^th^, 2022, one leaf located at the fifth node on four separate plants (2 plants from each plot) under each treatment were placed in a 2 x 3 cm chamber of a Li-COR 6400 (Li-COR Biosciences Inc. Lincoln, NE, USA). The leaf temperature was set to 23°C with a relative humidity of 60–70% and a CO_2_ level held at 800 µmol mol^-1^. Measurements were performed on cloudy days to maximize the effect of supplemental lighting while minimizing the effect of natural sunlight. Leaves were kept in the chamber until a steady-state photosynthetic rate was obtained, then the readings taken over a two-minute period were averaged.

### 2.4 Chlorophyll fluorescence

On January 27^th^, 2022, pepper leaves were selected based on location and exposure to supplemental lighting and dark-adapted for 20 minutes using aluminum foil. They were then placed in 6 cm^2^ chamber of the Li-COR 6800 fitted with the fluorometer head. The minimum fluorescence in a dark-adapted stated (F_o_) was collected once fluorescence stabilized after which an 800ms saturating light pulse (8000 µmol m^-2^ s^-1^) of red light was emitted to obtain the maximum fluorescence (F_m_). From F_o_ and F_m_, the variable fluorescence in a dark-adapted state (F_v_) was calculated (F_v_=F_m_-F_o_) to then determine the maximum efficiency of photosystem II (PSII; F_v_/F_m_). Next, leaves were acclimated to an actinic light level of 400 µmol m^-2^ s^-1^ (360 µmol m^-2^ s^-1^ of red light and 40 µmol m^-2^ s^-1^ of blue light; approximately the light level during a cloudy day) until the fluorescence levels (F_t_) stabilized. Once static, leaves were subjected to another saturating light pulse (F’_m_) followed by a dark pulse (F’_o_; 25 µmol m^-2^ s^-1^ of far-red light). These measurements were used to calculate the efficiency of PSII photochemistry (ΦPSII=(F’_m_-F_t_)/F’_m_), photochemical quenching (qP=(F’_m_-F_t_)/(F’_m_-F’_o_)), non-photochemical quenching (NPQ=(F_m_-F’_m_)/F’_m_), and the linear electron transport rate (ETR=ΦPSII*PPFD*0.5 where PPFD is the absorbed light and 0.5 is a factor that accounts for partitioning of energy between the two photosystems).

### 2.5 Photosynthetic pigment analysis

On January 24^th^, three circular samples of approximately 1cm in diameter were taken from a selected leaf. Leaves were chosen based on positioning and exposure to supplemental lighting. Ten samples in total were collected for each light treatment, each from a separate leaf. The samples were immediately weighed and flash frozen in liquid nitrogen, and placed in a -80°C freezer until analysis. Samples were extracted in 1mL of 95% ethanol in a warm (50°C) water bath for three hours. The ethanolic fraction was removed and placed into a new tube. The sample was further extracted once more and both aliquots were combined for a total extract volume of 2 mL. After the extractions, the sample was devoid of green color, indicating that the photosynthetic pigment had been completely extracted. Samples were then analyzed at 664 nm, 649 nm, and 470 nm in a UV/Vis spectrophotometer (UV-1600PC. VWR. Mississauga, Ontario, Canada). Concentrations of chlorophyll *a*, *b*, and carotenoids were determined using the equations from (Lichtenthaler, 1987).

### 2.6 Carbohydrate analysis

Three circular discs of approximately 1 cm in diameter samples were taken from the fifth leaf on six separate plants under each lighting treatment. Leaves were chosen based on positioning and exposure to supplemental lighting. The samples were taken at 21:30 on January 27^th^, 2022 (PM measurement) and again at 5:30 on January 28^th^, 2022 (AM measurement). These time points represent the carbohydrate accumulation at the end of the day (PM measurements) and at the end of the night (AM measurements). Samples were immediately weighed and then flash frozen in liquid nitrogen and kept in an -80°C freezer until analysis.

Leaf samples were extracted with 1mL of 80% ethanol in a warm (50°C) water bath for one hour. The supernatant was removed, ensuring no tissue was disturbed, and placed in a clean vial. The procedure was repeated for a total of 3 times until the tissue was devoid of green pigment (Tetlow and Farrar, 1993). The 3mL of ethanolic fraction was then dried using a vacuum concentrator and the paled leaf tissue was kept for further starch analysis. The remains were reconstituted in deionized water and soluble sugars were assayed using a sucrose/fructose/glucose kit (Megazyme. Wicklow, Ireland). To analyze sucrose, the samples were added to PMMA cuvettes and mixed with β-fructosidase and incubated at room temperature for 5 minutes. Deionized water, a buffer solution, and NADP^+^/ATP were then added to the cuvette and incubated for an additional 3 minutes at room temperature before analysis at 340 nm (A1_suc_). Hexokinase plus glucose-6-phosphate dehydrogenase was then added and incubated at room temperature for 5 minutes and a second reading was recorded at 340 nm (A2_suc_). The same procedure was repeated without the addition of tissue sample to obtain a blank (A1_sblank_ and A2_sblank_). For glucose and fructose assays, the sample was mixed in a PMMA cuvette with deionized water, a buffer solution, and NADP^+^/ATP and left to incubate for 3 minutes before analysis at 340 nm (A1_g+f_). Hexokinase plus glucose-6-phosphate dehydrogenase was then added to the sample, mixed, and was left to incubate at room temperature for 5 minutes before a second reading was taken at 340 nm (A2_g+f_). Lastly, phosphoglucose isomerase was added to the cuvette and incubated for 10 minutes at room temperature before a final analysis at 340nm (A3_g+f_). The same procedure was completed without any analyte to obtain blank values (A1_g+fblank_, A2_g+fsblank_, and A3_g+fblank_). Absorbance values were determined using the following equations:


$$A_{glucose}=\left(A2_{g+f}-A1_{g+f}\right)-\left(A2_{g+fblank}-A1_{g+fblank}\right)$$

$$A_{sucrose}=\left(\left(A2_{suc}-A1_{suc}\right)-\left(A2_{sblank}-A1_{sblank}\right)\right)-A_{glucose}$$

$$A_{fructose}=A3_{g+f}-A2_{g+f}$$

The content (C; mg g^-1^ of fresh weight (FW)) of each soluble carbohydrate was then calculated with the following:


$$C\left(mg\;g^{-1}FW\right)=\left(\frac{\left(\frac{V*MW}{\epsilon *d*v}\right)*A}{c}\right)$$

Where V is the final volume of the solution, MW is the molecular weight of the carbohydrate being analyzed (i.e., glucose, fructose, or sucrose), ϵ is the extinction coefficient of NADPH at 340 nm, d is the light path (cm), v is the sample volume, A is the absorbance of the carbohydrate being analyzed (i.e., A_glucose_, A_fructose_, or A_sucrose_), and c is the concentration of the ethanolic extract (g mL^-1^).

To assay starch, paled tissue after ethanolic extraction was lyophilized overnight and ground in a homogenizer before suspension in sodium acetate (100 mM). Thermostable α-amylase was then added to the sample. The sample was vortexed then placed in a boiling water bath for 15 minutes and was periodically vortexed throughout. The sample was then placed in a 50°C water bath for 5 minutes to equilibrate the temperature. Amyloglucosidase was then added to the sample, vortexed, and incubated in a warm (50°C) water bath for 30 minutes. The sample was removed from the warm water bath and left to cool at room temperature for 10 minutes. The sample was then centrifuged at 13,000 rpm for 5 minutes. A subsample of the supernatant was mixed with sodium acetate buffer and vortexed to create a stock solution. The stock solution was mixed with GOPOD reagent, incubated at 50°C for 20 minutes then analyzed in a spectrophotometer at 510 nm (A_s_) in PS cuvettes. A blank was obtained using the same procedure without the tissue sample (A_b_). Starch content was calculated using the following equation:


$$Starch\;\left(g\;100mL^{-1}\right)=\;(A_{s}-A_{b})*F*\frac{DV}{SV}*0.9$$

Where F is the absorbance value of glucose, DV is the diluted sample volume, and SV is the sample volume taken for analysis. The starch content was then converted to mg g^-1^ FW using the weight of the sample taken before the tissue was frozen.

### 2.7 Antioxidant analysis

The antiradical activity in pepper leaves was determined based on a modified version of a previously reported method (Alrifai et al., 2020). Three leaf samples from six separate leaves under each lighting treatment were taken on February 11^th^, 2022, weighed, and then flash frozen in liquid nitrogen and placed in a -80°C freezer. Before performing the analysis, the tissue was lyophilized overnight. Freeze-dried tissue was ground in a homogenizer and a subsample was transferred into a new microcentrifuge tube. The subsamples were homogenized further and 1 mL of 100% methanol was added to each microfuge tube. The samples were then left on a nutator overnight at room temperature. The next morning, the samples were centrifuged at 13,000 rpm for five minutes. The supernatant was collected in a clean tube before suspending the pellet in 1mL of fresh 100% methanol. Again, the sample was placed on a nutator for three hours before being centrifuged and having the supernatant removed. Both supernatant fractions were mixed together in a single tube and placed in a -20°C freezer until analysis. Fresh 2,2-diphenyl-1-picrylhydrazly (DDPH; 350 µM) was prepared immediately before analysis. In a cuvette, 1 mL of DPPH was mixed with 125 µL of methanolic sample extract and placed in the dark to incubate for 30 minutes before the absorbance was measured at 517 nm. The procedure was completed in duplicate. A standard curve was prepared in quadruplicate using the same assay technique but replacing the methanolic sample extract with ascorbic acid (AA; 0.025 mM-1 mM concentrations). All samples were expressed as µg AA mg^-1^ FW.

The ferric reducing antioxidant power (FRAP) assay of pepper leaves was determined using a modified version of a previously reported method (Alrifai et al., 2020). FRAP reagent was prepared fresh and consisted of 300 mM acetate buffer (pH 3.6), 20 mM FeCl_3_, and 10 mM 2, 4, 6-Tris (2-pyridyl)-s-triazine (TPTZ) in 40 mM HCl. 100 µL of methanolic sample extract was mixed with 900 µL of FRAP reagent and incubated on a heat block at 37°C for 1 h before reading the absorbance at 593 nm. A standard curve was completed using the same assay technique but ascorbic acid (AA; 0.025 mM-0.25 mM concentrations) was used instead of the tissue sample. All samples were expressed as µg AA mg^-1^ FW.

### 2.8 Yield

Pepper harvest began on November 23^rd^, 2021 and continued weekly until April 5^th^, 2022. Peppers were harvested once they had reached full maturity and had gone through a 75% color change.

### 2.9 Statistical analysis

All statistics were performed using SAS Studio 3.5. After the analysis of variance (ANOVA), multiple means comparisons between the different treatments were done using a Tukey-Kramer adjustment and a value of p<0.05 to indicate a significant difference. The greenhouse experiment was a randomized complete block design with 2 replications. Regression analysis was done using a backward elimination method. Final regressions with a p<0.05 were determined to be significant.

## 3 Results

Plants grown under the 100B treatment had significantly longer internodes than the control (0B) treatment (20.9% increase, **Table 2**). Plants grown under 150B did not have a increase in internode length and were similar to plants under 0B. The length and width of the 5th leaf as well as the stem diameter measured at the 5^th^ internode were statistically similar. The percent leaf dry matter (p=0.096) and specific leaf weight (SLW; p=0.057) were also similar under all light treatments (**Table 2**).

**Table 2:** Internode length, length and width of the 5^th^ leaf, stem diameter, dry matter percentage of the fifth leaf and specific leaf weight (SLW) of pepper cv. ‘Gina’ measured on January 24^th^, 2022 under four different lighting treatments.

Treatment	Internode Length (cm)	5^th^ Leaf Length (cm)	5^th^ Leaf Width (cm)	Stem Diameter (mm)	% Leaf Dry Matter	SLW (g m^-2^)
0B	4.39 ± 0.20^B^	17.8 ± 0.7^A^	9.9 ± 0.5^A^	8.44 ± 0.32^A^	13.4 ± 0.3^A^	20.1 ± 0.7^A^
50B	4.51 ± 0.34^AB^	17.9 ± 1.0^A^	10.6 ± 0.5^A^	8.53 ± 0.32^A^	14.7 ± 0.5^A^	17.8 ± 1.2^A^
100B	5.31 ± 0.18^A^	18.8 ± 0.9^A^	11.4 ± 0.4^A^	9.03 ± 0.31^A^	14.7 ± 0.5^A^	19.5 ± 1.2^A^
150B	4.69 ± 0.17^AB^	16.0 ± 0.5^A^	10.0 ± 0.2^A^	8.55 ± 0.30^A^	14.6 ± 0.3^A^	16.8 ± 0.6^A^

Mean values +/- standard error are representative of n=8. Within each parameter, different letters indicate significant differences as determined by a one-way ANOVA with a Tukey-Kramer adjustment (p<0.05).

**Figure 2:**
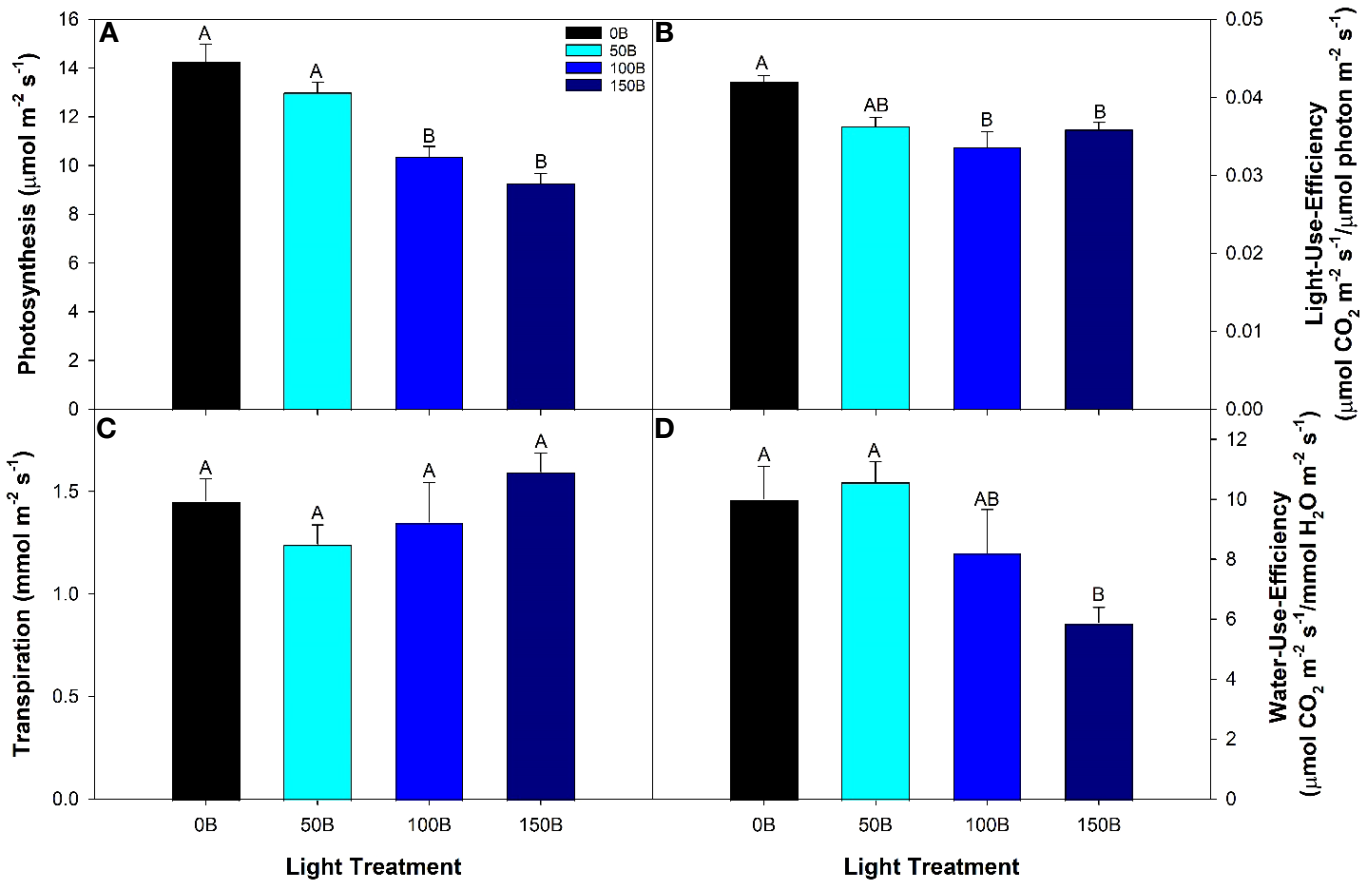
Photosynthesis **(A)**, light-use-efficiency **(B)**, transpiration **(C)**, and water-use-efficiency **(D)** of the 5^th^ leaf of pepper cv. ‘Gina’ grown under the different lighting treatments. Measurements were performed with a Li-COR 6400 fitted with a 2 x 3 cm clear top chamber on a cloudy day and thus represent the parameters mostly driven by the supplemental lighting. Mean values +/- standard error are representative of n=4. Within each parameter, different letters indicate significant differences as determined by a one-way ANOVA with a Tukey-Kramer adjustment (p<0.05).

Daytime photosynthetic rates from the 50B treatment were similar to the control (0B) treatment, but both 100B and 150B treatments had reduced photosynthetic rates when compared to both 0B and 50B (**Figure 2A**). Since the intrinsic supplemental lighting intensity was different between all four treatments, the photosynthetic rate was normalized on the incoming light intensity (both supplemental and natural) that each leaf was subjected to (i.e., light-use-efficiency). After normalization, leaves under the 50B light treatment still fixed similar amounts of CO_2_ per photon as leaves under the 0B treatment. Again, leaves under both 100B and 150B treatments produced lower light-use-efficiencies than leaves under the control (**Figure 2B**). In all treatments, the amount of water loss due to transpiration was similar (**Figure 2C**). Accordingly, in leaves under the 150B treatment water-use-efficiency was lowest.

**Figure 3:**
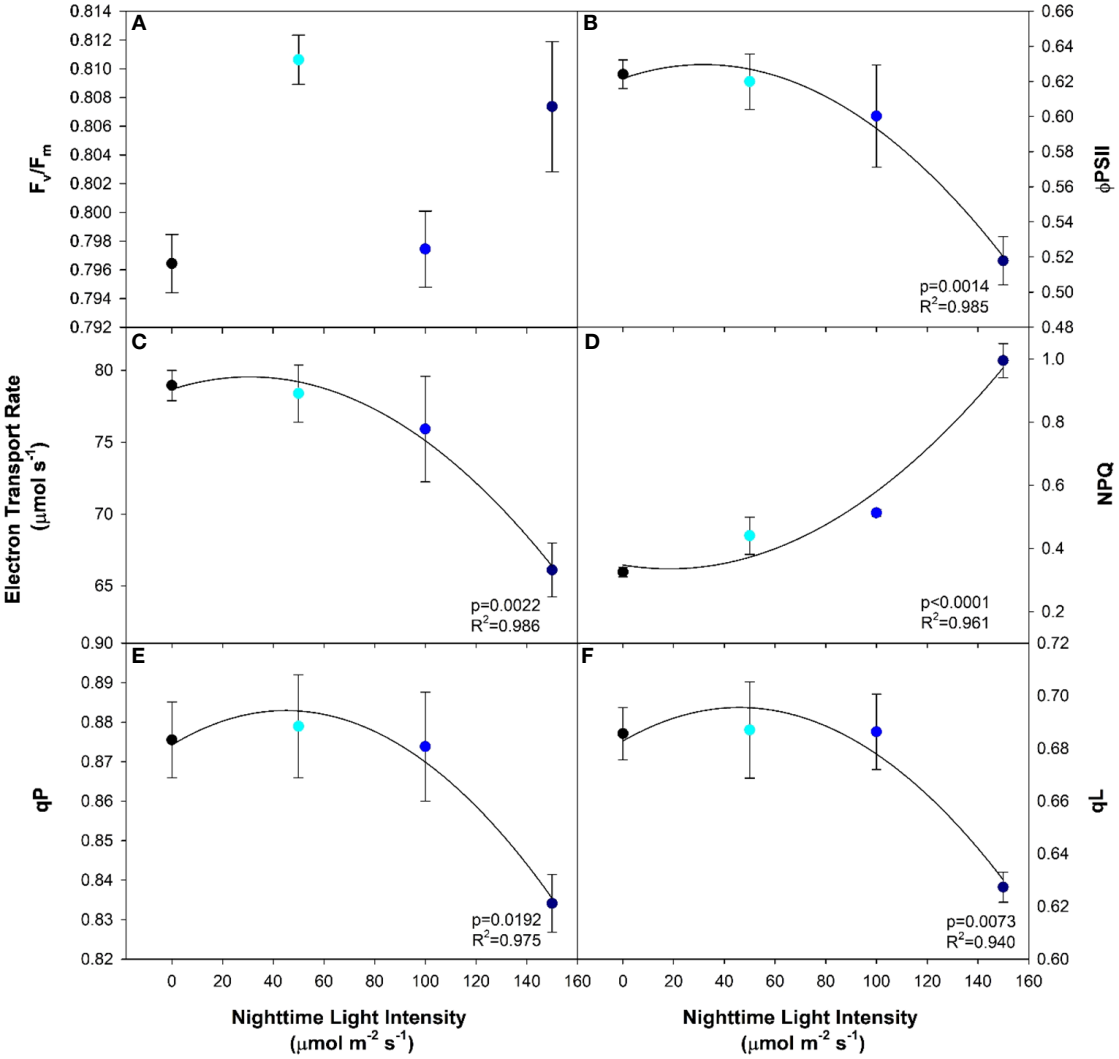
Chlorophyll fluorescence parameters including maximum efficiency of photosystem II in the dark-adapted state (F_v_/F_m_; **(A)**), the efficiency of photosystem II chemistry in the light-adapted state (ΦPSII; **(B)**), the electron transport rate (ETR; **(C)**), the non-photochemical quenching (NPQ; **(D)**), photochemical quenching (qP; **(E)**), and fraction of PSII reaction centers which are open (qL; **(F)**) from pepper leaves cv. ‘Gina’ grown under all light treatments. Regression analysis was completed using the backwards elimination method. Each data point represents the mean +/- the standard error of n=4. Only significant (p<0.05) regression analyses are represented in the graphs with p-values found in the bottom right corner of each respective graph.

T1he maximum efficiency of photosystem II (PSII) in the dark-adapted state (F_v_/F_m_) is typically used to identify stress in the plant caused by the light treatment (Kitajima and Butler, 1975; Baker, 2008). In this study, leaves under all light treatments had the same F_v_/F_m_ indicating that no photoperiod-related injury occurred even under the highest nighttime blue light intensity (**Figure 3A**). In contrast to the results from the dark-adapted measurements, PSII operating efficiency in the light-adapted state (ΦPSII) was observed to significantly decrease with increasing nighttime light intensity after exposure to a 400 µmol m^-2^ s^-1^ actinic light (**Figure 3B**). This was most-likely an attempt at photoprotection by means of inactivation of PSII reaction centers. The inactivation of PSII subsequently led to a decrease in linear electron transport rate (ETR); a phenomenon that was also observed as the nighttime light intensity increased (**Figure 3C**). As the inactivation of PSII increases with nighttime light intensity, a growing amount of incoming radiation must be dissipated in order to protect the leaf. One way that excess light energy is dispelled is through non-photochemical quenching (NPQ) which is achieved through thermal dissipation. Therefore, as PSII inactivation occurs, NPQ increases with increasing nighttime light intensity (**Figure 3D**). Subsequently, as NPQ increases and ΦPSII decreases, photochemical quenching (qP) as well as the fraction of open PSII reaction centers (qL) also decreased (**Figures 3E, F**). Although dark-adapted measurements showed no photoperiod related injury, when we consider all the above information, it is clear that as the nighttime light intensity increased, the biochemical use of the incoming radiation shifted from usage in the light reactions to energy dissipation via NPQ in an effort to protect the photosynthetic machinery.

**Figure 4:**
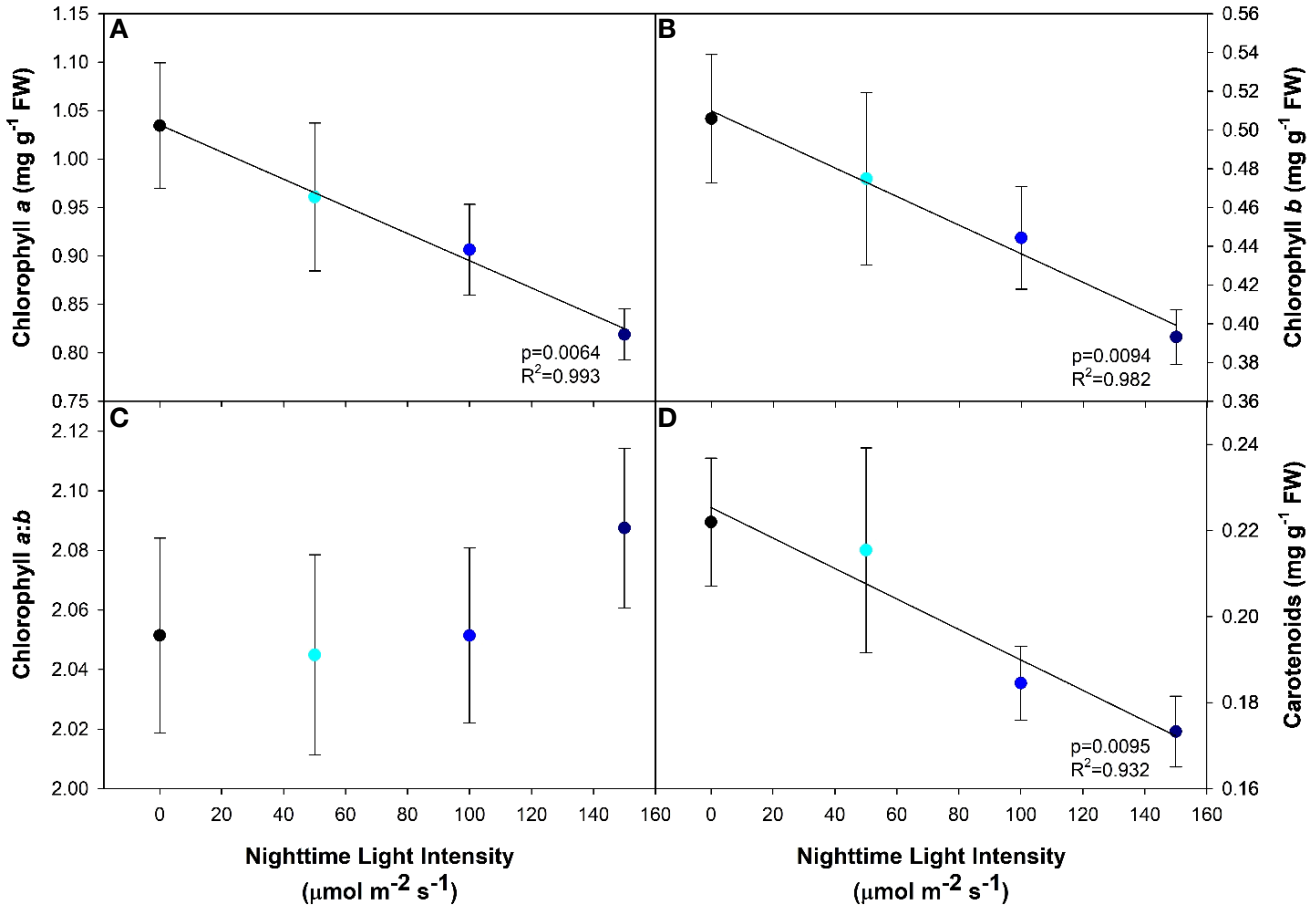
Photosynthetic pigment concentrations of pepper cv. ‘Gina’ leaves grown under all light treatments. Chlorophyll *a* **(A)**, chlorophyll *b* **(B)**, chlorophyll *a*:*b* **(C)** and carotenoids **(D)** from leaf samples. Regression analysis was completed using the backwards elimination method. The data points represent the mean +/- standard error of n=10. Only significant (p<0.05) regression analyses are represented in the graphs with p-values found in the bottom right corner of each respective graph.

Both chlorophyll *a* and *b*, and to a lesser extent carotenoids, are important pigments which funnel light into the photosynthetic pathway. Generally speaking, the higher the photosynthetic pigment concentration, the more light the plant will be able to capture and utilize. In our study, we observed a decreasing linear relationship between all three photosynthetic pigments with increasing nighttime blue light intensity (**Figures 4A, B, D**). In contrast, the ratio of chlorophyll *a* to *b* was unaffected by the light treatments (**Figure 4C**).

**Figure 5:**
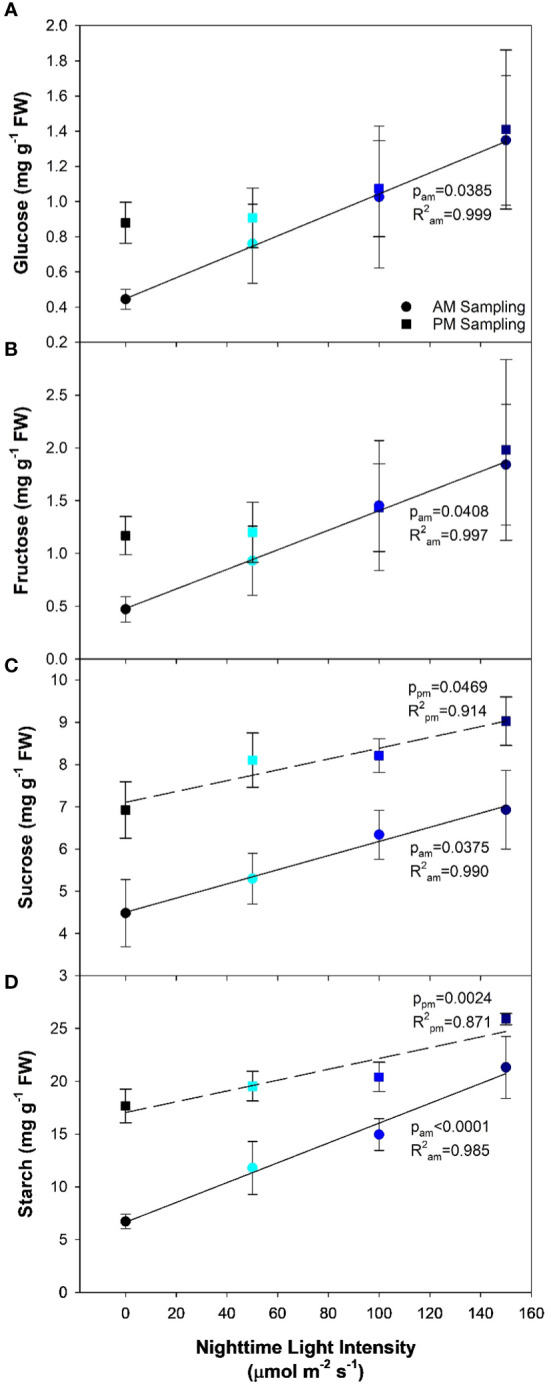
Glucose **(A)**, fructose **(B)**, sucrose **(C)**, and starch **(D)** concentrations from the analysis of the 5^th^ leaf of pepper cv. ‘Gina’ plants representing each of the 4 lighting treatments. The leaves were sampled twice, once in the AM (5:30) and once in the PM (21:30). The AM sampling represents what happens to the carbohydrate profile during the night period while the PM sampling represents what happens during the daytime. Regression analysis was completed using the backwards elimination method. Mean values +/- the standard error are representative of n=6. Only significant (p<0.05) regression analyses are represented in the graphs with the coloring of the regression line and p-value corresponding to the sampling time.

Analysis of leaf carbohydrates provides insight in to the leaf’s ability to produce and export the end product of photosynthesis. Here, we observed that as the nighttime light intensity increased, so too did the concentration of soluble carbohydrates (i.e., glucose, fructose, and sucrose; **Figures 5A–C**) as determined during the AM sampling period. Starch, which is mainly thought of as a storage molecule, typically degrades during the night period to support the carbon needs of the plant. However, as the nighttime light intensity increased, starch levels in the leaf remained high; an indication that starch was not being converted at the same rate as in plants that had an 8h dark period (0B; **Figure 5D**). In fact, the starch level in plants grown under 150B was almost four times higher than observed in 0B plants.

PM sampling measurements represent the accumulation of carbohydrates during the day period. Both glucose and fructose showed no significant differences among light treatments (**Figures 5A, B**). Leaves grown under 0B had drastically increased glucose and fructose concentrations compared to the AM sampling, as would be expected. Interestingly, as the nighttime light intensity increased, a difference between the concentrations of glucose and fructose during the PM sampling and AM sampling was nearly non-existent. In all CL treatments (50B, 100B, and 150B), the daytime light intensity was reduced proportionally to keep the DLI similar to the control (0B). Therefore, it would be expected that lower amounts of carbohydrates would be synthesized compared to plants grown under the 0B treatment during the daytime. However, the similar soluble sugar concentrations during the AM and PM sampling indicate a lack of movement of these carbohydrates during the night period revealing a potential bottleneck in carbon metabolism. Lastly, concentrations of both sucrose and starch were observed to increase as the nighttime light intensity increased in the PM sampling (**Figures 5C, D**).

**Figure 6:**
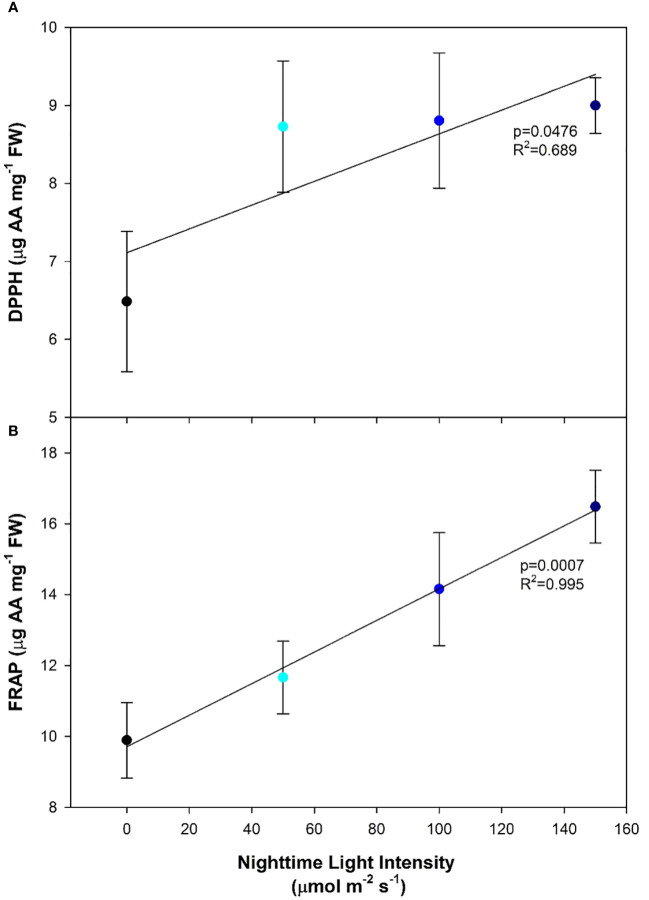
Antioxidant activity levels of pepper cv. ‘Gina’ leaves measured by 2,2-diphenyl-1-picrylhydrazyl (DPPH; **(A)**) and ferric reducing antioxidant power (FRAP; **(B)**) grown under various light treatments. DPPH is expressed as µg of ascorbic acid (AA) mg^-1^ of fresh weight. Regression analysis was completed using the backwards elimination method. Mean values +/- the standard error are representative of n=6.

Antioxidants are produced to inhibit oxidation and the production of free radicals; both of which can damage the cell. DPPH radical-scavenging activity was observed to increase as the nighttime light intensity increased (**Figure 6A**). Similarly, the ferric reducing antioxidant power (FRAP) was also observed to increase with increasing nighttime light intensity (**Figure 6B**). This indicates that plants under higher nighttime light intensities were under more oxidative stress than those which had lower or no nighttime lighting (**Figure 6**).

**Figure 7:**
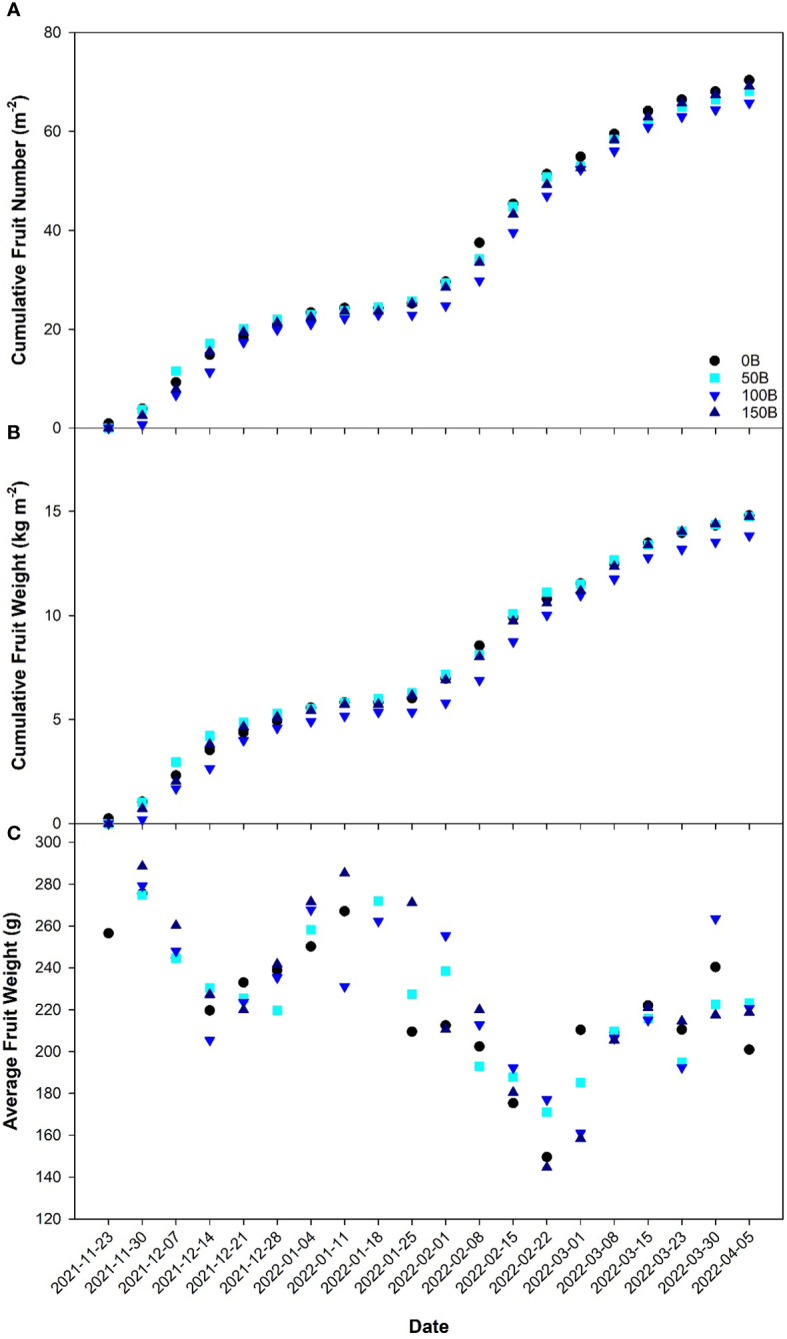
Cumulative fruit number **(A)**, cumulative fruit weight **(B)**, and average fruit weight **(C)** of pepper cv. ‘Gina’ grown under all light treatments as recorded weekly from November 23^rd^, 2021 to April 5^th^, 2022.

Cumulative fruit number (**Figure 7A**) and cumulative fruit weight (**Figure 7B**) followed very similar trends throughout the 20-week harvest period and were unaffected by the light treatments indicating that CL treatments (50B, 100B and 150B) can sustain crop yield similar to the 16 h control (0B). During the initial harvest period, the average fruit weight was high in all light treatments (**Figure 7C**). Throughout the remainder of the experiment, while average fruit weight tended to oscillate, the general trend was that fruit size decreased in all treatments.

## 4 Discussion

### 4.1 Dynamic CL with monochromatic blue light sustains plant growth in peppers

With sustainability driving many innovations in the agricultural space, the implementation of low intensity, long photoperiod lighting strategies (including CL) has received much interest as a way to shift electricity usage to the off-peak, nighttime hours (Velez-Ramirez et al., 2012; Haque et al., 2015; Hao et al., 2018; Lanoue et al., 2019; Lanoue et al., 2021a, b). During photoperiod lengthening or CL, the circadian rhythm of the plant is often disrupted due to the lack of synchronization between the endogenous periodicity and exogenous environmental stimuli (Velez-Ramirez et al., 2017b). This asynchrony leads to a down regulation of photosynthesis accompanied by leaf chlorosis and a reduction in yield. However, CL could increase plant biomass and yield if injury were to be prevented (Velez-Ramirez et al., 2012; Lanoue et al., 2019).

The results presented in this paper indicate that pepper plants were able to grow under supplemental CL with a nighttime light intensity of up to 150 µmol m^-2^ s^-1^ of blue light without obvious visual injury. While most morphological parameters were unchanged by growth under CL, it is notable that plants grown under 100B had increased internode length when compared to the 16 h control (20.9% increase, **Table 2**). Conversely, Demers et al., 1998b showed that broad spectrum CL from HPS lamps caused shorter pepper plants. However, in our study, monochromatic blue light was used during the night period and when used as a sole source, blue light can increase stem elongation (Hernández and Kubota, 2016; Lanoue et al., 2019; Kong and Zheng, 2020). The increased stem elongation can aid in reducing fruit stacking brought about by short internodes with supplemental lighting which may cause misshapen fruit, impacting quality (Lanoue et al., 2022b). A further increase in internode length was not observed when the nighttime light intensity was raised to 150 µmol m^-2^ s^-1^. Under a low level of monochromatic blue light, phototropin is activated and this can promote stem elongation (Kong and Zheng, 2020). However, as the blue light intensity increases, a shift to higher activation rates of cryptochrome occurs, inhibiting stem elongation (Kong and Zheng, 2020). Although the light intensity at which this change in blue light photoreceptor activation occurs is unknown, it is clear that levels above 100 µmol m^-2^ s^-1^ of sole blue light did not further increase stem elongation. It should be noted that other methods to increase internode length thereby averting fruit stacking does exist. The increase in the difference between the day and night temperature (typically facilitated by the increase of the daytime temperature) has been observed to have a positive impact on stem elongation (Carvalho et al., 2002). Additionally, the introduction of far-red lighting into a supplemental lighting strategy can also increase stem elongation. Additional far-red light will shift the phytochrome photo-stationary state in favor of phytochrome being in the inactive form which in turn stimulates stem and internode elongation (Demotes-Mainard et al., 2016). Both of these strategies would also elicit an increase in internode length similar to that observed in the 100B treatment. However, the blue light played a dual role in meeting the DLI requirement for photo-assimilate and biomass production and promoting proper morphological change.

Throughout the experiment, the yield and fruit size were similar regardless of which light treatment the pepper plants were grown under (**Figure 7**). Notably, fruit quality as measured by Brix analysis was unaffected by CL treatments (data not shown) which indicates that elevated carbohydrate levels in the leaves of plants grown under CL did not translate to increased sugar levels in the fruit. Previous research has shown that the utilization of a drastically lower nighttime light intensity compared to the daytime can reduce the chlorotic damage caused by CL (Matsuda et al., 2016; Velez-Ramirez et al., 2017b; Lanoue et al., 2019; Pham and Chun, 2020; Lanoue et al., 2021b). In contrast, our previous study utilized a similar nighttime light intensity (147 ± 3 µmol m^-2^ s^-1^) but with broad spectrum white light and observed chlorosis and yield reduction in peppers (Lanoue et al., 2022b). Since both studies used pepper plants and the nighttime light intensities were almost identical, the conflicting results can be explained by the light spectra used. However, it should be noted that the pepper cultivar used in these two studies were different and therefore a genotype-dependent response could also be possible. These studies indicate that to avoid CL-injury, a reduction in nighttime light intensity or a change in spectral spectrum is needed. If CL is to be successful in averting injury and maintaining yield, a dynamic strategy with changes in light intensity, or light spectrum or both between daytime and nighttime must be employed.

The circadian rhythm of the plant is altered by the use of CL which can cause changes in the expression of proteins and enzymes. Notably, *type III light harvesting chlorophyll a/b binding protein 13* (*CAB-13*) has been identified as a key player in CL-injury in tomato (Velez-Ramirez et al., 2014). However, the circadian rhythm complexes are intricate, allowing for many possible points of regulation (Inoue et al., 2018). Utilizing a dynamic spectral shift during CL (i.e., a change in the light spectra between the day and night) has shown promise in reducing CL-injury and increasing yield in several crops (Matsuda et al., 2016; Ohtake et al., 2018; Lanoue et al., 2019). Implementing dynamic CL as opposed to maintaining a continuous intensity of broad spectrum light, has the potential to partially restore the plant’s natural circadian rhythm, aiding in injury prevention. In fact, Velez-Ramirez et al., 2017b found that dim blue light (10 µmol m^-2^ s^-1^) during the night following a white light spectrum also reduced CL-injury in tomato. Furthermore, our previous research on peppers and tomatoes has shown that up to 75 µmol m^-2^ s^-1^ of blue light during the night did not cause CL-injury (Lanoue et al., 2019; Lanoue et al., 2021b). Therefore, the switch from white light during the day to blue light at night may be able to maintain circadian synchrony allowing for proper gene expression of *CAB-13*.

The current understanding related to blue light averting or preventing injury is poorly understood. Regarding spectral quality, photoreceptors become a natural area of interest to understand the interaction with circadian entrainment (Marie et al., 2022); in the case of blue light, specifically cryptochrome. While not entraining the circadian rhythm itself, cryptochrome can interact with other protein regulators which have known to impact on the core circadian clock (Shor et al., 2017). Under continuous blue light, the circadian rhythm of leaf movement is naturally entrained to a 24h period, whereas exposure to continuous darkness, green light, red light, or far-red light resulted in a phase shift away from a 24h period (Halaban, 1969). The incidence of blue light can stabilize cryptochrome which can determine the phase of the circadian rhythm (He et al., 2022). In the case of the current research and previous literature (Halaban, 1969; Lanoue et al., 2019; Lanoue et al., 2022a), blue light was able to entrain the circadian rhythm to 24h, simulating a natural 24h period involving light and dark periods (such as 16h light, 8h darkness). The interaction between blue light and cryptochrome has also been postulated to regulate circadian clock associated 1 (CCA1) expression. It was found that in plants deficient in cryptochrome 1 and 2, an arrhythmic circadian rhythm was observed, again indicating that cryptochrome plays an important role in entrainment (Mo et al., 2022). Mo et al. (2022) postulated that a possible mechanism for circadian entrainment was the blue light input loop, in which blue light activated a down stream effect, mediated through cryptochrome. However, the exact mechanism has yet to be determined and therefore requires further research.

### 4.2 Continuous lighting, photosynthetic feedback, and photochemistry

The constant photosynthetic pressure from CL led to increased carbohydrate levels compared to plants grown under the 16 h photoperiod (0B), regardless of nighttime light intensity (**Figure 5**). Demers et al., 1998b observed higher levels of starch in pepper leaves grown under CL compared to a 14 h photoperiod, whereas soluble sugars were unaffected. Due to this high carbohydrate accumulation, an inevitable increase in ROS production also occurred, which can be attributed to the over-reduction of electron acceptors under constant light pressure (Velez-Ramirez et al., 2011; Zha et al., 2019; Kumar et al., 2022). Similar to other studies (Haque et al., 2015; Zha et al., 2019; Wen et al., 2021; Kumar et al., 2022) the antioxidant capacity increased under CL and displayed a significant linear relationship with increasing nighttime light intensity (**Figure 6**). This increase in DPPH and FRAP activity suggests an increased need from the plant for ROS-scavenging in order to prevent further oxidative stress to the photosynthetic machinery as well as DNA damage (Huang et al., 2019). In fact, the increase in ROS-scavenging ability may be partially responsible for the lack of chlorosis observed in peppers as similar responses have been observed in lettuce, a species which is CL-tolerant (Wen et al., 2021). Similar increases in ROS-scavenging were observed by Haque et al., 2017 when a variable temperature strategy was used during CL to mitigate injury. Consequently, we hypothesize that high nighttime blue light intensities (150 µmol m^-2^ s^-1^) are causing a hermetic/adaptive effect, which may be absent when the light spectrum remains constant (Jalal et al., 2021). This allows for the plant to manage the elevated ROS levels through increased ROS-scavenging abilities thus limiting photosynthetic reduction (Huang et al., 2019).

Together with the increase in antioxidant capacity, a subsequent reduction in photosynthetic pigments was also observed. As the nighttime light intensity increased, chlorophyll *a*, chlorophyll *b*, and carotenoid content decreased (**Figure 4**). In peppers, the literature is inconclusive regarding the relationship between photosynthetic pigments and CL. Some literature suggests that chlorophyll content in peppers was negatively correlated with the lengthening of the photoperiod (Dorais, 1992), while others found that chlorophyll was unaffected by CL (Murage and Masuda, 1997). Conversely, our DLI was 34% higher than that used by Murage and Masuda, 1997 which could account for the disparities seen between the two studies.

When plants are exposed to excess light or an environment with a fluctuating light intensity, they must be able to cope with the abiotic stress while maintaining efficient light harvesting processes to avoid photodamage (Kaiser et al., 2015). In the case of CL, radiation pressure is constant and can elicit plant stress responses. Similar to an increase in antioxidants, a decrease in photosynthetic pigments, such as chlorophyll, can aid in the reduction of photo-oxidative stress caused by excess light when carbohydrate levels are increased. By reducing the amount of chlorophyll pigment, and coincidentally the antenna size/efficiency (Jin et al., 2016), less light energy would be transferred to the primary electron acceptor. Collectively, the reduction in chlorophyll content and the production of ROS-scavenging enzymes represent acclimation responses to the constant photon pressure of CL to mitigate further oxidative stress of PSII.

Without question, growth under CL can cause plants to undergo a stress response. However, the extent to which the abiotic factor causing the stress response becomes harmful to the plant as opposed to beneficial is difficult to quantify. A common metric used to measure plant stress is assessing the maximum efficiency of PSII using F_v_/F_m_ (Bilger et al., 1995; Baker, 2008; Janka et al., 2015; Guidi et al., 2019). F_v_/F_m_ is quantified using a dark-adapted leaf, and therefore measures the reaction center of PSII in the open state (i.e., when the primary acceptor quinine is fully oxidized). In this way, we believe this measurement places the plant in an artificial state which is not representative of normal growth conditions. Our study shows that even under CL with high nighttime light intensity of 150 µmol m^-2^ s^-1^, F_v_/F_m_ was unaffected when compared to plants grown under 0B; a traditional 16 h photoperiod. In a light-adapted state, PSII operating efficiency at the actinic light intensity (ΦPSII) and quenching coefficients (NPQ and qP) as well as ETR can be determined. A contrasting narrative unfolded when employing light adapted metrics. Although the actinic light level was identical during measurements of all treatments (400 µmol m^-2^ s^-1^), ΦPSII, ETR, qP, and qL are observed to decrease with increasing light intensity while NPQ is seen to increase (**Figure 3**). This suggests that when measured in a dark-adapted state, leaves appear to be without injury, while during light-adapted measurements, PSII was unable to perform optimally as the nighttime light intensity increased. Since NPQ is used as a photoprotective mechanism to preserve PSII (Ruban et al., 2007; Ruban and Murchie, 2012), it then stands to reason that as the nighttime light intensity increased, plants exhibit reduced capability to utilize incoming light in the photosynthetic process and are protecting themselves from the light they are exposed to. In an effort to alleviate photodamage of PSII, it is possible that there was an uptick in cyclic electron flow around PSI (Yamori and Shikanai, 2016). In this instance, an imbalance would be created between ATP and NADPH formation, creating a large proton gradient needed for NPQ, and have downstream implications on carbon metabolism (Joliot and Johnson, 2011). In contrast to a decrease in photosynthetic pigment and increase in antioxidant capacity, PSII photochemistry is an instantaneous response to current conditions (Muller et al., 2001). With the underlying acclimation response, light-adapted chlorophyll fluorescence measurements were able to elucidate the real-time response of leaves to incoming radiation. Together, a reduction in photosynthetic pigments, increase in ROS-scavenging, and increase in NPQ aimed to mitigate the harmful effects of excessive radiation, in this case, CL, during plant growth. While the photosynthetic rate and LUE were reduced as nighttime light intensity increased (**Figure 2**), reductions in LUE were minimized due to their coping mechanisms thus yield in peppers was unaffected.

One suggested mechanism for the observed impact of nighttime intensity on light-adapted measurements, while dark-adapted measurements remain unaffected, is the rate of ribulose 1,5-biphosphate (RuBP) regeneration (Baker, 2008). In *Phaseolus vulgaris* L. leaves which had artificially elevated carbohydrate levels due to sucrose treatment, RuBP regeneration was observed to be the limiting factor in photosynthetic performance (Araya et al., 2006). In the present study, the carbohydrate levels when plants were grown under all CL treatments were observed to be higher than in plants grown under treatment 0B which involved an 8h dark period (**Figure 5**). The presence of increased carbohydrate under CL coincided with reduced photosynthesis and LUE (**Figures 2A, B**). Furthermore, it was observed that carbohydrate levels remained unaltered (glucose and fructose) or only marginally decreased (sucrose and starch) after the nighttime period (AM sampling) in plants grown under CL compared to the PM sampling (**Figure 5**). The lack of carbohydrate loss during the night period was similar to that found in tomatoes grown under CL (Demers and Gosselin, 2002; Haque et al., 2015). This shows a disconnect between the light intensity, photosynthetic rate, carbon export, and carbohydrate status of the leaf. Typically, carbon export, the process by which soluble carbohydrates are moved out of the leaf, increases with light intensity (Jiao and Grodzinski, 1996) and daytime export is always higher than nighttime export due to the presence of light (Lanoue et al., 2018). Here, we observed elevated leaf carbohydrate levels even when the nighttime light level was 150 µmol m^-2^ s^-1^ suggesting an imbalance between source and sink tissue activity. Since light is present, but export seemed to be lacking, enzymes related to the export pathway may be under circadian control (Chincinska et al., 2013) similar to those in the sucrose biosynthetic pathway (Jones and Ort, 1997). With a build-up of carbohydrates in the leaves, but yield being sustained in the current CL treatments, examination of the link between carbon export and CL is of interest in potentially realizing yield gain under such a lighting strategy.

## 5 Conclusion

Although increased carbohydrate content and ROS-scavenging capability as well as decreased photosynthetic pigment content signal a potential adverse response to CL, it was not observed to impair the yield of pepper plants in this study. Furthermore, based on the commonly used stress metric F_v_/F_m_, measured in a dark adapted state, all plants grown under CL treatments displayed low levels of stress, similar to the 0B treatment. Interestingly, although dark-adapted chlorophyll fluorescence measurements were unaffected by light treatment, light-adapted chlorophyll fluorescence measurements seemed to be impacted. The data implies that ΦPSII and ETR decreased while NPQ increased with increasing nighttime light intensity. In dark-adapted chlorophyll fluorescence measurements, the saturating light pulse is meant to occur fast enough that photosynthesis will not be initiated. While dark-adapted measurements do not drive photosynthesis, light-adapted measurements incorporate feedback about the downstream photosynthetic products. Light adapted measurements integrate the feedback that carbohydrate levels are elevated and respond with a reduction in ΦPSII, ETR, and qP and an increase in NPQ. Therefore, we suggest the use of light-adapted chlorophyll fluorescence measurements may be a more appropriate method in identifying stress in CL tolerant crop-types.

## Data availability statement

The original contributions presented in the study are included in the article/**Supplementary Material**. Further inquiries can be directed to the corresponding author.

## Author contributions

JL: Conceptualization, Data curation, Formal Analysis, Investigation, Methodology, Writing – original draft, Writing – review & editing. SS: Data curation, Investigation, Writing – review & editing. CL: Data curation, Investigation, Writing – review & editing. XH: Conceptualization, Funding acquisition, Investigation, Methodology, Project administration, Supervision, Writing – review & editing.
