# Supplementary material for: Photosynthetic adaptation strategies in peppers under continuous lighting: insights into photosystem protection
Source: Front Plant Sci. 2024 May 31;15:1372886. doi: 10.3389/fpls.2024.1372886 (PMC11176547; doi:10.3389/fpls.2024.1372886)
Supplement: Supplementary file 1 [file Image_1.pdf]

Supplementary Figure

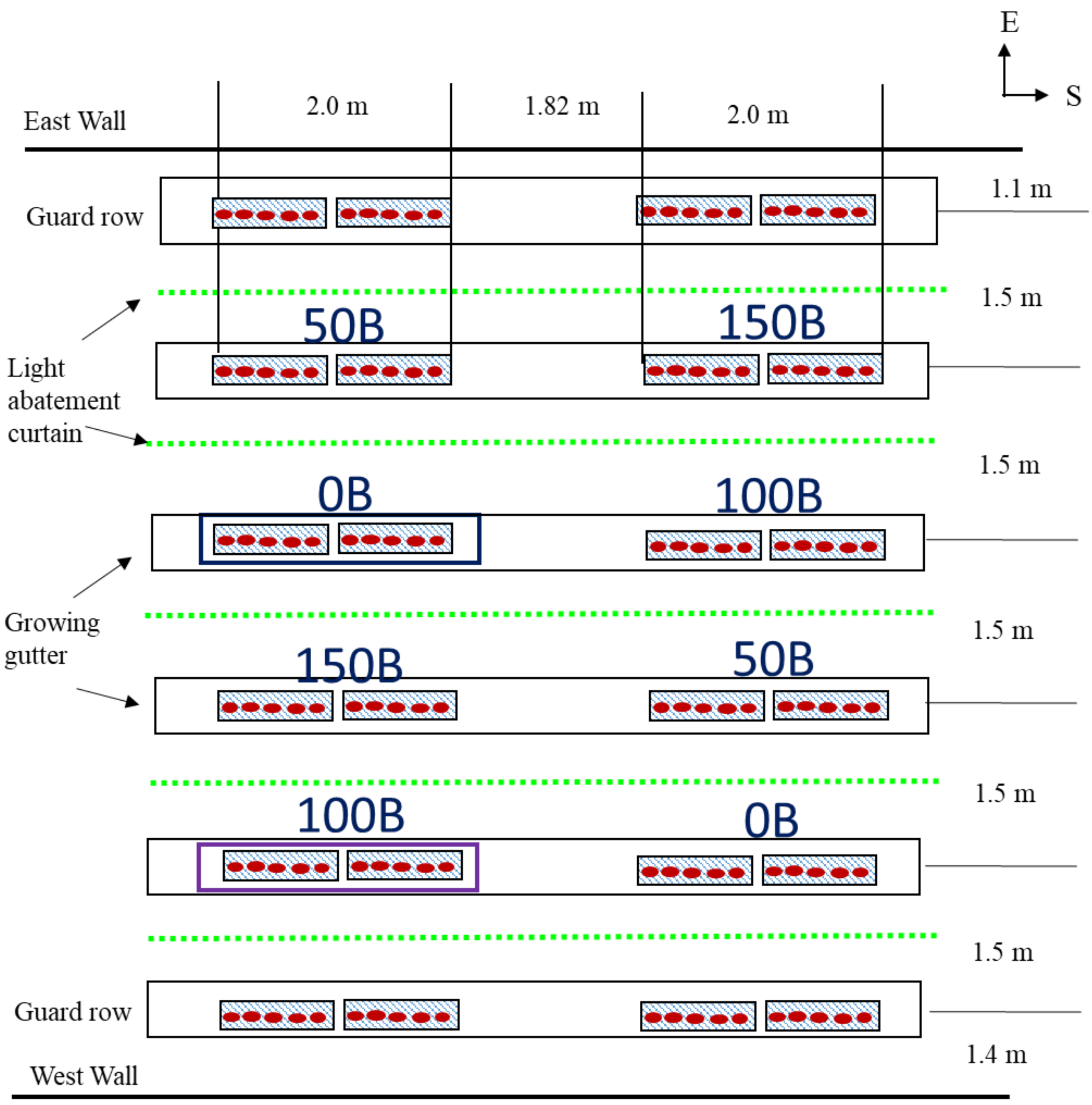

Supplementary Figure 1. Layout of the greenhouse pepper lighting experiment
